# Dosimetric assessment of rigid setup error by CBCT for HN‐IMRT

**DOI:** 10.1120/jacmp.v11i3.3187

**Published:** 2010-05-28

**Authors:** Danielle Worthy, Qiuwen Wu

**Affiliations:** ^1^ Department of Radiation Oncology Wayne State University Detroit Michigan 48201 USA; ^2^ Department of Radiation Oncology Duke University Medical Center Durham North Carolina 27710 USA

**Keywords:** IMRT, head‐and‐neck cancer, setup error, margin

## Abstract

Dose distributions in HN‐IMRT are complex and may be sensitive to the treatment uncertainties. The goals of this study were to evaluate: 1) dose differences between plan and actual delivery and implications on margin requirement for HN‐IMRT with rigid setup errors; 2) dose distribution complexity on setup error sensitivity; and 3) agreement between average dose and cumulative dose in fractionated radiotherapy. Rigid setup errors for HN‐IMRT patients were measured using cone‐beam CT (CBCT) for 30 patients and 896 fractions. These were applied to plans for 12 HN patients who underwent simultaneous integrated boost (SIB) IMRT treatment. Dose distributions were recalculated at each fraction and summed into cumulative dose. Measured setup errors were scaled by factors of 2–4 to investigate margin adequacy. Two plans, direct machine parameter optimization (DMPO) and fluence only (FO), were available for each patient to represent plans of different complexity. Normalized dosimetric indices, conformity index (CI) and conformation number (CN) were used in the evaluation. It was found that current 5 mm margins are more than adequate to compensate for rigid setup errors, and that standard margin recipes overestimate margins for rigid setup error in SIB HN‐IMRT because of differences in acceptance criteria used in margin evaluation. The CTV‐to‐PTV margins can be effectively reduced to 1.9 mm and 1.5 mm for CTV1 and CTV2. Plans of higher complexity and sharper dose gradients are more sensitive to setup error and require larger margins. The CI and CN are not recommended for cumulative dose evaluation because of inconsistent definition of target volumes used. For fractionated radiotherapy in HN‐IMRT, the average fractional dose does not represent the true cumulative dose received by the patient through voxel‐by‐voxel summation, primarily due to the setup error characteristics, where the random component is larger than systematic and different target regions get underdosed at each fraction.

PACS numbers: 87.53.Kn, 87.53.Tf.

## I. INTRODUCTION

Head and neck (HN) cancer is often treated with intensity‐modulated radiation therapy (IMRT) for its capability to provide highly conformal dose distributions to target volumes and improve sparing of normal tissue structures.^(^ ^1^ ^–^ ^2^ ^)^ The improved conformity of IMRT plans is typically demonstrated by the steep dose gradients present in the dose distribution. Compared to 3D conformal radiotherapy (3DCRT), sharp dose gradients are believed to make IMRT dose distributions more sensitive to geometric uncertainties, such as interfraction setup errors.^(^ ^3^ ^)^ This is a major concern, as the delivered dose to the patient may deviate significantly from the approved plan, not only preventing the benefits of IMRT over 3DCRT from being realized clinically, but also potentially resulting in delivery of a worse treatment.

In order to ensure that the adequate doses are delivered accurately to tumor volumes, geometric uncertainties need to be taken into account by adding a margin to the clinical target volume (CTV) to create a planning target volume (PTV).^(^ ^4^ ^–^ ^5^ ^)^ Optimal treatment delivery requires that margins are defined properly such that tumor volumes receive adequate dose while simultaneously minimizing damage to surrounding healthy tissues. With respect to patient interfraction setup errors, there are a number of margin recipes which have been reported and reviewed in the literature. These recipes can be used to calculate planning margin size using systematic (Σ) and random (σ) setup errors measured for a population of patients. Of the margin recipes proposed, two commonly used are those from van Herk et al.^(^ ^6^ ^)^ (M1=2.5Σ+0.7σ) and Stroom et al.^(^ ^7^ ^)^ (M2=2.0Σ+0.7σ). Both recipes were derived and validated based on analysis of 3D conformal treatments, and their validity in HN‐IMRT planning is still being investigated. One primary goal of this work is to evaluate the dose deviations due to the rigid setup errors on simultaneous integrated boost (SIB) HN‐IMRT treatments to determine appropriate margins.

Several works have reported the effects of setup errors on HN‐IMRT treatments.^(^ ^3^ ^,^ ^8^ ^–^ ^14^ ^)^ For most of these works, patient setup errors were ‘simulated’ with positional displacements either arbitrarily chosen or derived from a Gaussian distribution.^(^ ^3^ ^,^ ^9^ ^,^ ^12^ ^–^ ^14^ ^)^ This makes the assumption that there is no variation in random errors for a patient population. Review of measured setup deviations in HN‐IMRT patients shows that this assumption is not valid^(^ ^15^ ^)^ and, therefore, the setup errors used are not truly representative of the patient setup error distribution.

In this work, setup errors were not simulated, but measured from a substantial population (30 patients) using daily cone‐beam computed tomography (CBCT) imaging. Application of measured setup errors to HN‐IMRT dose distributions were reported previously in a few studies. For example, Lawson et al.^(^ ^11^ ^)^ measured setup errors for 12 patients using daily orthogonal kV X‐ray images, Hong et al.^(^ ^10^ ^)^ used a high precision optically guided localization system for 10 patients; and O'Daniel et al.^(^ ^16^ ^)^ used an integrated CT‐linac system for 11 patients. Comparatively, CBCT localization offers advantages over orthogonal image pair and optical guidance systems, such as an improved accuracy of measurement from 3D CT images, as well as a greater number of parameters (i.e. rigid and nonrigid misalignments) that can be determined.^(^ ^17^ ^–^ ^18^ ^)^ Additionally, while O'Daniel et al. did use CT imaging to measure patient setup errors, images were only taken twice per week throughout treatment and therefore the clinical setup error distribution used may not have been well represented.^(^ ^16^ ^)^ In this study, we also investigated topics that have not been explored previously. These include analysis on the influence of dose distribution complexity to rigid setup errors, and investigation in the consistency of calculated dose delivered to the patient in the presence of setup errors, using two different computation methods – cumulative and average fractional summation.

## II. MATERIALS AND METHODS

### A. Patient data and treatment plans

Twelve simultaneous integrated boost (SIB) HN‐IMRT cases were randomly selected for this study. For each HN‐IMRT case, two types of plans were created using the gradient‐based optimizers: ideal fluence optimization (FO) and direct machine parameter optimization (DMPO), both available within the ${\text{Pinnacle}}^{3}$ treatment planning system (Phillips Radiation Oncology Systems, Madison WI). The DMPO plans were clinically deliverable plans. The FO does not take into account the machine delivery constraints and yields dose distributions that have superior plan quality,^(^ ^19^ ^)^ are more conformal and have steeper dose gradients; therefore, it represents an ideal situation. The purpose of evaluating these two plans was to study the effect of setup errors on dose distributions with different dose gradients.

Details on the targets, dose prescriptions, planning objectives and treatment plans used in this study were presented in a previous report.^(^ ^19^ ^)^ Briefly, the gross tumor volume, GTV1, encompassed the primary tumor and involved lymph nodes; a 5 mm margin was used for expansion to the clinical target volume, CTV1. A second clinical target volume, CTV2, included all electively treated lymph nodes. For both target volumes, a 5 mm CTV‐to‐PTV margin was used. Organs‐at‐risk (OARs) included the brainstem (BS), spinal cord (SC), mandible (M) and parotid glands (PG). Planned treatments consisted of 35 fractions. The prescription dose was 70 Gy to PTV1 and 60 Gy to PTV2. Planning objectives were: 1) $V_{\text{Rx}}$ ≥90% for targets, where $V_{\text{Rx}}$ represents the target volume percentage receiving the prescription dose; 2) $D_{1}<$ tolerances for OARs (45 Gy for the SC, 54 Gy for the BS, 65 Gy for M), where the $D_{1}$ value represents the dose delivered to 1% of the OAR volume, or the maximum dose; and 3) to minimize parotid gland doses.

### B. Rigid setup error characterization and analysis

The patients are immobilized with aquaplast mask secured to an IPPS carbon fiber extension board (CIVCO, Orange City, IA). CBCT imaging was performed daily, and manual registration of the CBCT to the planning CT based on bony anatomy in the CTV1 region is done for each fraction and yields 3D setup error data in the form of an isocenter shift in the x (left‐right (LR)), y (anterior‐posterior (AP)), and z (superior‐inferior (SI)) directions. Only translational setup errors were used in this study. For 30 patients (including the 12 patients described in Section II. A), daily CBCT setup error data was measured. For some patients, CBCT data was not available for all 35 treatment fractions. The setup error data from a total of 896 CBCTs, or an average 30 CBCTs per patient, were collected and analyzed.

Statistical analysis was conducted and setup error was characterized^(^ ^20^ ^)^ for these patients from CBCT measurement. For patient, *i*, the mean, $\mu_{i}$, and standard deviation, $\sigma_{i}$ of the isocenter shifts in the x, y and z directions were calculated from all fractions. Setup error data for the patient population was characterized by four parameters: the mean of each patient specific mean M($\mu_{i}$), the standard deviation of each patient specific mean, $\Sigma (\mu_{i})$, the root‐mean‐square of each patient specific standard deviation, $\text{RMS}(\sigma_{i})$, and the standard deviation of each patient specific standard deviation, $\Sigma (\sigma_{i})$. A detailed discussion of these parameters has been previously reported.^(^ ^20^ ^)^

The effect of rigid setup errors was investigated by recalculating the dose distribution on the planning CT following translational shifts of the patient at each fraction with respect to the accelerator coordinate system. For each of the 12 patient treatment plans, dose distributions were recomputed on the planning CT associated with each plan for all 896 treatment fractions for which setup errors were measured. Cumulative dose distributions were then calculated for each of the 30 patient treatments. Considering that for some patient treatments, setup error data were not available for all fractions, the cumulative dose was estimated in the following manner. For each treatment fraction, *k*, for patient *i*, the dose to each voxel is calculated to be $D{\left(x,y,z\right)}_{i,k}$. Assuming equal contribution, each voxel dose is normalized by the total number of fractions, *n*, for which setup error data are collected. The cumulative dose distribution for patient *i*, is then calculated as a voxel‐by‐voxel summation of the doses for each fraction, *k*, over all fractions, *n*, shown below

(1) $$D{\left(x,y,z\right)}_{i}=\frac{35}{n}\sum_{k=1}^{n}D{\left(x,y,z\right)}_{i,k}$$

### C. Dosimetric evaluation criteria

Evaluation of the dosimetric effects on target coverage and OAR sparing was conducted using dose indices provided by dose volume histograms (DVHs). Dose indices assessed for target volumes included $D_{m}$, the mean dose, $D_{95}$, $D_{90}$ and $D_{50}$ (the dose to 95%, 90% and 50% of the target volume, respectively), and $V_{\text{Rx}}$, the relative target volume receiving prescription dose. For OARs, $D_{m}$ and $D_{1}$ were evaluated for the brainstem, spinal cord and mandible, while for the parotid glands, $D_{m}$, as well as $V_{30}$ and $V_{20}$ (the volumes receiving 30 and 20 Gy, respectively) were assessed.

For the static dose treatment plans (i.e. plans without any setup errors), there is a degree of variability in the results for the same dose index amongst these 12 patients. To facilitate comparison between patients, the dose indices from each fraction or cumulative dose distributions were normalized to their respective values from the static plan. With respect to target volumes, accurate dose delivery to the CTV is the primary goal of treatment. Based on the ICRU formalism, giving a planned dose to the static PTV ensures that the prescribed dose is delivered to the CTV under motion.^(^ ^4^ ^–^ ^5^ ^)^ Therefore, to assess whether the CTV received a dose equal to its planned value, CTV dose metrics at each fraction or cumulative doses were normalized with respect to the PTV value in static plan.

In addition to dose metrics, conformity index (${\text{CI}}_{\text{RI}}$) and conformation number (${\text{CN}}_{\text{RI}}$) were calculated to determine setup error effects on dose distribution conformity to the primary tumor volume (CTV1). The conformity index is defined by:^(^ ^5^ ^)^

(2) $$CI_{RI}=\frac{V_{RI}}{TV}$$

where $V_{\mathrm{RI}}$ represents the tissue volume covered by the reference isodose (RI) and *TV* represents the target volume. Ideal conformation is achieved when the ${\text{CI}}_{\text{RI}}$ equals 1. Values greater than 1 correspond to excess irradiation volume outside of the target volume. Values less than 1 indicate partial target volume irradiation. The conformation number^(^ ^21^ ^–^ ^22^ ^)^ is defined as:

(3) $$CN_{RI}=\frac{TV_{RI}}{TV}\times \frac{TV_{RI}}{V_{RI}}$$

where ${\mathrm{TV}}_{\mathrm{RI}}$ is the target volume that receives the reference isodose. This index combines two indices considered separately by Lomax and Schieb,^(^ ^23^ ^)^ called the target coverage conformity index (first fraction) and the healthy tissues conformity index (second fraction). The range of values for all three indices – target coverage, healthy tissues and conformation number – is between 0 (no conformation to the target) and 1 (perfect conformation to the target). For both the ${\text{CI}}_{\text{RI}}$ and the ${\text{CN}}_{\text{RI}}$, the reference isodoses used in calculation were 100%, 95% and 90% of the prescription dose for PTV1. Both the CI and CN were calculated using PTV1 as the target volume for the plan, while CTV1 was used for the actual treatments.

### D. Margin evaluation

Margins based on published recipes^(^ ^6^ ^–^ ^7^ ^)^ and the measured patient setup error data were compared to the overall 5 mm CTV‐to‐PTV margins currently used in the planning. It should be emphasized that in this work only margins for rigid setup uncertainties were investigated. Additional margins are necessary for other uncertainties such as tumor shrinkage and non‐rigid setup errors, and should be included in the overall planning margin.

Previously, margin adequacy has been investigated by replanning patient treatments with variable PTV margins when geometric uncertainties such as setup errors are introduced.^(^ ^8^ ^,^ ^13^ ^)^ In this study, an alternative approach was used. Margin adequacy for DMPO plans was investigated using a constant margin value (5 mm), and measured setup errors were scaled up by a factor of 1–4 (referred to as scale 1, scale 2, etc.). Dose distributions were then recalculated for each scaled dataset. Normalized dose metrics, as well as CI and CN indices, were used for the evaluation. If planning margins for target volumes are optimal, then normalized cumulative dose metrics will have a value of 1. This ensures that the planned dose coverage is achieved for the CTV volumes. Inadequate target coverage and breakdown of margins was noted when normalized dose index values for targets fell below 1.

### E. Dose distribution sensitivity evaluation

The dosimetric effects of rigid setup errors on dose distributions with different degrees of complexity were also evaluated in this study. As mentioned above, two types of plans – ideal FO and deliverable DMPO – were analyzed. Dosimetric effects were investigated by applying scale 4 setup errors to each type of dose distribution, and recomputing the cumulative dose. A similar analysis of normalized dose metrics, conformity index and conformation number were also performed. Additionally, dose gradients were calculated for DMPO and FO static (planned) and treatment dose distributions. Dose gradients within the entire tissue volume were estimated as the ratio of conformity index values with different reference isodoses, specifically the ratio of ${\text{CI}}_{95}$ and ${\text{CI}}_{90}$ to ${\text{CI}}_{100}$ (i.e. ratio of the tissue volumes receiving 95% to 100% of the PTV1 prescription dose and 90% to 100% of the PTV1 prescription dose). Results are indicative of the relative size of each dose volume with respect to the prescription dose volume within the dose distribution. Greater ratios represent larger distances between dose volumes and shallower dose gradients. Smaller ratios, closer to a value of 1, indicate highly conformal dose volumes and steeper dose gradients. Similarly, for the primary tumor volume, ratios of the target coverage conformity index, specifically, the ratios of ${\text{TV}}_{\left(90,95,100\right)}/\text{TV}$ were computed as an indicator of gradient sharpness within the tumor volume.

Margins necessary for FO plans could not be determined in the same manner as for DMPO plans as dose distributions were only recalculated for scale 4 setup errors. Therefore, they were estimated in the following way. It was assumed that DMPO and FO results differ by a constant factor, $DVH_{\frac{FO}{DMPO}}$, which can be calculated as

(4) $$DVH_{\frac{FO}{DMPO}}=\frac{DVH_{FO}}{DVH_{DMPO}}$$

where ${\mathrm{DVH}}_{\mathrm{FO}}$ and ${\mathrm{DVH}}_{\mathrm{DMPO}}$ represent the normalized DVH index results for FO and DMPO treatment dose distributions. Factors were calculated for each of the dosimetric indices used in evaluation of CTV1 and CTV2 target volumes and subsequently were applied to the DMPO normalized DVH results obtained for setup error with scales 1–3. This gives an estimate of the FO normalized DVH values at other scales of setup error from which margin breakdown was inferred.

### F. Cumulative dose vs. fractional dose

Another objective of this work was to determine whether the average fractional dose is representative of the actual cumulative dose due to setup errors that is delivered to the patient. For DMPO plans, the influence of margin size on dose deviations calculated using these two analyses methods were studied by applying two magnitudes of setup error, scale 1 and scale 4, representative of large and small margins, respectively. Cumulative and average fractional analyses and summation were then performed for each patient case. For cumulative analysis (CUMU), the cumulative dose was calculated as described in Section II.B, with evaluation based on the cumulative DVH. In contrast, for average fractional analysis (AVG), for a given SIB HN‐IMRT plan, a daily DVH was computed for each fraction, *k*, that setup errors were collected within treatment of each patient,*i*. Subsequently, for each patient treatment, dosimetric indices calculated from each daily DVH were averaged over all treatment fractions, *n*, yielding the average fractional DVH used for evaluation in this study. Results for both analyses methods were compared using normalized dose metrics.

## III. RESULTS

### A. Static plan dose characteristics

It is of interest to first describe the dose characteristics of the static DMPO and FO patient plans before discussing the setup error effects on the delivered dose distribution. In an earlier work, both DMPO and FO plans were created for the same subset of SIB HN‐IMRT patients, and selected plan quality metrics for targets and OARs were compared.^(^ ^19^ ^)^ A summary of the average DVH dose metric results previously found is presented in Table 1, along with additional dose metric results for the mandible and parotid glands. As expected, FO plans have superior plan quality, with target volume coverage that exceeds planning goals and significantly lower OAR doses for critical structures. For DMPO plans, all OAR planning goals are met; however, target volume coverage falls short of planning goals with an average of 88%±3.5% of PTV1 and 85%±3.5% of PTV2 volumes receiving their respective prescription doses.

**Table 1:** Average select dosimetric index results over all 12 patients for DMPO and FO plans with standard deviation (SD) shown in parentheses. Average structure volumes are also presented for reference.

*Structure*	*DVH Index*	*DMPO (SD)*	*FO (SD)*	*Volume cc (SD)*
PTV1	VRx (%)	88.0 (3.5)	93.3 (2.5)	252.9 (132.9)
PTV2	VRx (%)	85.6 (3.5)	93.6 (3.0)	605.7 (321.9)
BS	D1 (Gy)	42.1 (3.0)	40.3 (3.5)	23.6 (9.3)
SC	D1 (Gy)	37.4 (0.6)	35.1 (1.9)	27.1 (9.3)
M	D1 (Gy)	65.6 (2.4)	65.4 (1.9)	76.6 (15.1)
PG	V30 (%)	47.8 (9.0)	44.3 (9.9)	61.2 (23.7)
PG	V20 (%)	69.5 (7.1)	64.2 (8.4)	–

BS=brainstem; SC=spinal cord; M=mandible; PG=parotid glands.

### B. Patient systematic and random error statistics

Table 2 presents statistics calculated to characterize the measured (scale 1) setup errors for the patient population used in this study. In all three directions, the magnitudes of the systematic and random errors are small (range: 1.3–2.6 mm), with systematic errors smaller than the random errors. Calculation of standard planning margins yield margin widths of M1 = 4.7−5.2 mm and M2 = 4.0−4.5 mm, depending on direction. These margins are very similar, likely due to the small systematic error results, and are comparable to the 5 mm CTV‐to‐PTV margin used in planning.

**Table 2:** Rigid setup errors measured by 3D CBCT analysis from 30 patients.

*Direction*	$M(\mu_{i})$	$\Sigma (\mu_{i})$	$RMS(\sigma_{i})$	$\Sigma (\sigma_{i})$
x: LR (mm)	−0.2	1.3	2.0	0.8
y: AP (mm)	0.3	1.3	2.6	1.2
z: SI (mm)	0.3	1.4	2.4	1.1

### C. Margin adequacy

**Figure 1:**
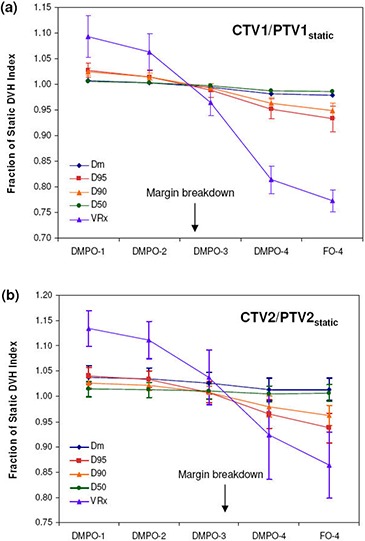
Target dose metrics for DMPO plans with setup error scales 1–4 and FO plans with setup error scale 4: (a) $\text{CTV}1/\text{PTV}1_{\text{static}}$; (b) $\text{CTV}2/\text{PTV}2_{\text{static}}$. Error bars are for one standard deviation.

**Figure 2:**
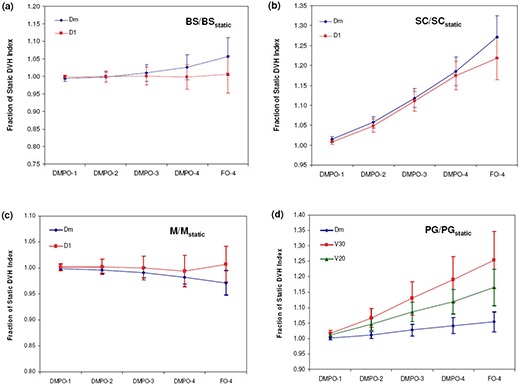
OAR dose metrics for DMPO plans with setup error scales 1–4 and FO plans with setup error scale 4: (a) brainstem (BS); (b) spinal cord (SC); (c) mandible (M); (d) parotid glands (PG). Error bars are for one standard deviation.

The dosimetric effects of measured setup errors (scale 1) on DMPO plans are presented in Fig. 1 for targets and Fig. 2 for OARs, with dose deviations averaged over all 12 patients. With respect to target volumes, dose coverage to CTV1 (Fig. 1(a)) and CTV2 (Fig. 1(b)) is not compromised when a 5 mm margin is used. This is indicated by all of the normalized target dosimetric indices having values greater than 1, and demonstrates that 5 mm planning margins can adequately handle the rigid interfractional motion encountered in the clinic. With regards to critical structures, estimated doses with setup errors are similar to those planned, with the greatest deviation being a 1.5% increase in $V_{30}$ to the parotid glands.

Figures 1 and 2 also present the dosimetric effect of larger setup errors (scales 2–4) on DMPO plans. In general, as the setup error magnitude increases, target coverage decreases and OAR doses increase. However, for the mandible (Fig. 2(c)) this is not the case. Instead, the $D_{m}$ values decrease while the maximum dose remains unchanged. The observed decrease in $D_{m}$ to the mandible may be associated with its proximity to, or overlap with, PTV1. Thus, as the dose to PTV1 decreases, the mean dose to the mandible also decreases. Of the 12 SIB HN‐IMRT patient cases used for planning, eight patients had significant overlap between the mandible and PTV1 volumes.

For CTV1, margin breakdown occurs for setup errors between scale 2 and scale 3, while for CTV2, breakdown occurs between scale 3 and scale 4. The exact margin failure point was estimated by linear interpolation of target dosimetric ratios as a function of setup error scale and was considered to be the approximate scale where all dosimetric ratios would fall below 1. For CTV1, this was a scale of 2.6 and for CTV2, a scale of 3.3 (excluding $D_{m}$ and $D_{50}$ which are fairly insensitive to setup errors). Since the setup error scale is a multiplicative factor, target margins can be estimated by dividing the 5 mm margin used in planning by each respective failure scale. Assuming that the setup error distribution from the 30 patients used in this study is representative of the patient population, target margins can be reduced to ~1.9 mm for CTV1(=5.0/2.6), and ~1.5 mm for CTV2(=5.0/3.3) in order to account for rigid setup errors adequately.

**Figure 3:**
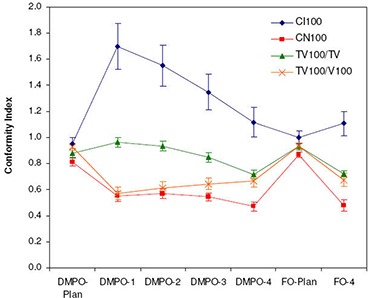
Target conformity of FO and DMPO plans and treatments. Indices presented include the ICRU conformity index (CI), conformation number (CN), target coverage conformity index (${\text{TV}}_{\text{RI}}/\text{TV}$) and healthy tissues conformity index $\left({\text{TV}}_{\text{RI}}/V_{\text{RI}}\right)$ for a 100% reference isodose.

**Figure 4:**
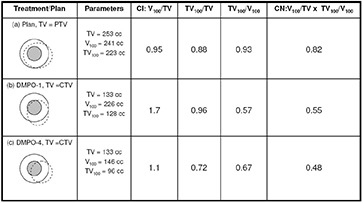
An example illustrating the limitations of conformity index (CI) and conformation number (CN) for a DMPO plan (a) and DMPO treatments with scale 1 (b) and scale 4 (c) setup errors applied for cumulative dose evaluation. Inner shaded small circle is CTV; outer circle is PTV; dashed line represents 100% isodose line.

Figure 3 summarizes the conformity index and conformation number results for DMPO plans and treatments for a 100% reference isodose. Similar trends were noted for the CI and CN results with 95% and 90% reference isodoses (not shown). Figure 3 shows that CI values are closest to 1 for DMPO plans (${\text{CI}}_{100}=0.95$) and significantly greater than 1 for DMPO treatments when scale 1 setup errors are applied (${\text{CI}}_{100}=1.7$). These results indicate excess irradiation volume outside of targets, and suggest that 5 mm margins can be reduced significantly and still maintain target conformity. The cause of the large unexpected increase in CI values for DMPO treatments compared to static plans stems from an inconsistency in the target volume of interest that is used for calculation (PTV1 for plan, CTV1 for treatments) and their difference in size (PTV1 is on average ~1.9 times greater than CTV1). This is illustrated in Fig. 4 with examples shown for the DMPO plan (a) and DMPO‐1 treatment (b). Essentially, isodoses are planned to cover PTV1 such that the irradiated volume in treatment matches CTV1. However, because of the large margin used, the irradiated volume actually delivered is much greater than CTV1 yielding the CI results observed. When larger scales of setup error are applied to DMPO treatments, CI values decrease toward 1; however, even with scale 4 setup errors, results never fall below 1, suggesting that the target is always covered. This contradicts normalized dose index results found earlier, where for scale 4 setup errors, the CTV1 volume was found to be partially covered compared to the static plan with an average normalized $V_{\text{Rx}}$ value of 0.81. Interestingly, this discrepancy is due to the inability of the CI to account for the degree of spatial intersection between the volume irradiated and the target volume, and is another major limitation to the use of CI as an evaluation parameter not only for plans but for treatments, as well.^(^ ^21^ ^)^ This limitation is also illustrated in Fig. 4 with an example shown for the DMPO‐4 treatment (c).

Average CN results are presented in Fig. 3 along with target coverage and healthy tissues conformity indices used in calculation of CN. Results for the DMPO plan are also closest to 1 (${\text{CN}}_{100}=0.81$), while values for DMPO treatments with scale 1 setup errors are significantly less than 1 (${\text{CN}}_{100}=0.55$). The poor CN values for treatments also stem from inconsistencies in the target volume chosen for evaluation and the differences in their size. As illustrated in Fig. 4, excess margins result in an irradiation volume much greater than the CTV1 volume used in treatment evaluation (b) and, subsequently, the very low healthy tissue conformity index values shown in Fig. 3. Additionally, with larger setup errors applied, CN values for DMPO treatments do not decrease but stay approximately constant. This is due to a balance between the target coverage conformity index and the healthy tissues conformity index. As larger setup errors are applied, CTV1 target coverage is reduced; however, this also reduces the healthy tissue volume irradiated, improving normal tissue sparing.

### D. Dose distribution sensitivity comparison

The average dose deviations at scale 4 setup errors for FO compared to DMPO dose distributions are also presented in Figs. 1 and 2. With regards to target volumes, FO treatments suffer greater dose deviations, with the greatest deviations observed for the $V_{\text{Rx}}$ index, with values of 0.77 and 0.86 for CTV1 and CTV2, respectively, compared to 0.81 and 0.92 for DMPO treatments. Critical structure dose deviations are also larger for FO treatments, especially with respect to the spinal cord (Fig. 2(b)), where the mean dose delivered for FO treatments was 1.27 times greater than planned vs. only 1.19 times greater in DMPO treatments.

Differences in the conformity of DMPO and FO plan and treatment dose distributions are presented in Fig. 3. As expected, FO plans have superior conformity, with mean ${\text{CI}}_{100}$ and ${\text{CN}}_{100}$ results of 1.0 and 0.87, compared to 0.95 and 0.81 for DMPO plans. However, this improvement in conformity is lost when setup errors are applied; instead, the conformity of DMPO and FO treatment dose distributions are comparable with ${\text{CI}}_{100}$ and ${\text{CN}}_{100}$ values of 1.11 and 0.48 for FO and 1.12 and 0.47 for DMPO.

The superior conformity of FO plans can only be achieved through sharper gradients within the dose distribution. As shown in Table 3, FO plans have sharper gradients both within and surrounding PTV1 (ratios are closer to 1). Unfortunately, these steep gradients cannot be realized in delivery in the presence of setup errors. Calculated differences in gradients between plan and treatment dose distributions are larger for FO than DMPO. The larger dose deviations observed in FO plans suggest that larger margin sizes are needed for more complex plans with sharper dose gradients.

**Table 3:** Dose gradient using CI and ${\text{TV}}_{\text{RI}}/\text{TV}$ for FO and DMPO plans and treatments. Differences are calculated between DMPO and FO treatments and their respective plans. Gradient values closer to 1 indicate steeper dose gradients within the dose distribution.

*Gradient*	*DMPO Plan*	*DMPO‐4*	*DMPO Difference*	*FO plan*	*FO‐4*	*FO Difference*
${\text{CI}}_{90}/{\text{CI}}_{100}$	2.23	2.92	0.69	1.77	2.94	1.17
${\text{CI}}_{95}/{\text{CI}}_{100}$	1.35	1.72	0.37	1.26	1.70	0.44
${\text{TV}}_{90}/\text{TV}/{\text{TV}}_{100}/\text{TV}$	1.13	1.36	0.23	1.07	1.35	0.28
${\text{TV}}_{95}/\text{TV}/{\text{TV}}_{100}/\text{TV}$	1.12	1.27	0.15	1.07	1.26	0.19

**Figure 5:**
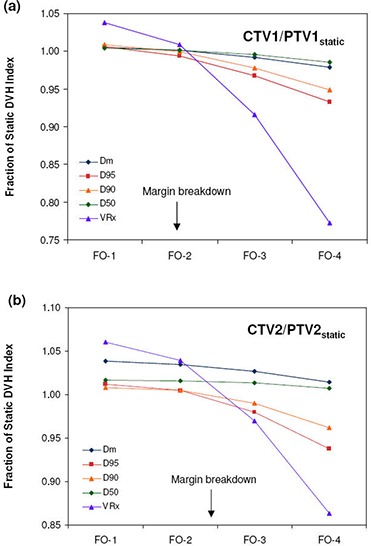
Estimated target dose metrics for FO plans with setup error scales 1–4: (a) $\text{CTV}1/\text{PTV}1_{\text{static}}$; (b) $\text{CTV}2/\text{PTV}2_{\text{static}}$. Error bars are for one standard deviation.

Figure 5 presents the estimated FO normalized DVH values at scales of setup error (scales 1–4) for both target volumes. The margin failure point was estimated in the same manner as for DMPO plans and was found to be approximately 1.9 for CTV1 and 2.4 for CTV2. These correspond to margin widths of 2.7 mm for CTV1 and 2.1 mm for CTV2, approximately 0.8 mm and 0.6 mm larger than the optimal DMPO margins.

### E. Cumulative dose vs. average fractional dose

**Figure 6:**
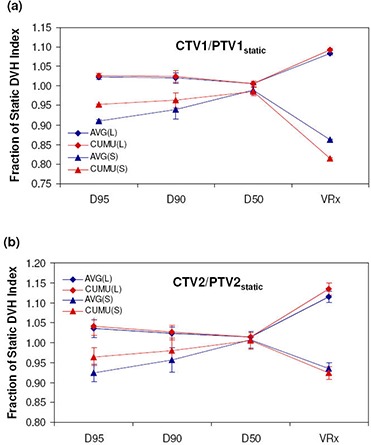
Target dose metrics for DMPO treatments calculated using AVG and CUMU methods with large (L) and small (S) margins: (a) $\text{CTV}1/\text{PTV}1_{\text{static}}$; (b) $\text{CTV}2/\text{PTV}2_{\text{static}}$.

**Figure 7:**
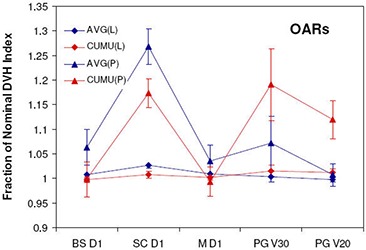
OAR dose metrics for DMPO treatments calculated using AVG and CUMU methods with large (L) and small (S) margins. BS=brainstem; SC=spinal cord; M=mandible; PG=parotid glands.

Results for average fractional and cumulative dose due to setup errors with large and small margins and averaged over all 12 patient plans are presented in Fig. 6 for targets and Fig. 7 for OARs. For both AVG and CUMU methods, the mean dose is exactly the same by definition; therefore, it is omitted in both Figs. 6 and 7. For the dosimetric index, $D_{50}$, also representative of the average dose to tissue volumes, ratios calculated by AVG and CUMU methods show very small difference, the largest being 0.3% for CTV1 with small margins. With respect to dosimetric indices more sensitive to setup errors, AVG results significantly deviate from CUMU results.

When small margins (scale 4 setup error) are used, AVG results for targets are lower for $D_{95}$ (−4.5%), and $D_{90}$ (−2.6%). For OARs, AVG doses are higher for the brainstem (+6.5%), spinal cord (+8.1%) and mandible (+4.2%) maximum doses ($D_{1}$), respectively. However, for the parotid gland dose volume indices, $V_{30}$ and $V_{20}$, AVG results are lower, both with differences of 11.0%. A similar trend is observed for larger margins (scale 1 setup error), with smaller differences and greater consistency (smaller standard deviation among patients) in normalized dosimetric results for both methods compared to results with small margins. For targets, all normalized dose indices predicted by the AVG method are lower with differences of −0.5% for $D_{95}$, −0.4% for $D_{90}$ and for $V_{\text{Rx}}$, −1.0% for CTV1 and −1.6% for CTV2. Maximum doses predicted by the AVG method for brainstem, spinal cord and mandible are higher by +1.0%, +1.9%, and +0.7%, respectively, while parotid normalized dose volume indices for $V_{30}$ and $V_{20}$ are −1.2% and −1.4% lower.

## IV. DISCUSSION

In this study, we retrospectively applied rigid setup errors measured from 30 patients to 12 SIB HN‐IMRT plans and estimated the dosimetric effects. Based on standard margin recipes,^(^ ^6^ ^–^ ^7^ ^)^ a 5 mm PTV margin should be optimal. Our results demonstrated that this margin was too large and could effectively be reduced to ~1.9 mm for CTV1 and ~1.5 mm for CTV2 and still provide adequate dose coverage. Overestimation of PTV margins by margin recipes for SIB HN‐IMRT has been reported elsewhere;^(^ ^12^ ^)^ however, specific causes for this discrepancy have not been discussed in detail. Comparatively, the most significant differences between these published margin recipes and this study are the treatment site investigated and the evaluation criteria used. In the works for margin recipes, prostate cancer treatment was studied and margins were selected to ensure that the minimum dose ($D_{\text{min}}$) or dose to 99% of the CTV volume ($D_{99}$) was greater than or equal to 95% of the prescription dose.^(^ ^6^ ^–^ ^7^ ^)^ This criterion can be reasonably met in the treatment of prostate cancer, which is a deep seated tumor, with a target volume that is generally of convex shape and is surrounded by few critical structures, mainly the bladder and rectum. For HN cancer, this criterion can not be easily met. This is because HN treatment is more complex due to the superficial location of tumors, the usually concave shape of the target volume and the close proximity of many critical structures. Therefore, a different criterion is commonly used, and margins were chosen based on $D_{90}$ to ensure that 90% of the target volume receives the prescription dose. This is considerably relaxed compared to the minimum dose requirement for prostate and may contribute to the significantly smaller margins required. This study demonstrates that the published recipes, although commonly used, should not be applied blindly to any tumor site without a thorough dosimetric study. In addition, margin width is also a function of the dose gradient/penumbra of the plan. Generally speaking, the margins for IMRT plans, with higher dose gradient, should be larger than the ones in 3DCRT.

It should be noted that in this work, an offline correction strategy was used to manage patient setup. This is reflected in the smaller systematic errors presented in this study of 1.3–1.4 mm as compared to other published works, where errors on the order of 2.0 mm were observed (range: 1–3 mm).^(^ ^15^ ^–^ ^16^ ^,^ ^24^ ^–^ ^26^ ^)^ This is an important point, as offline correction strategies have been shown to significantly reduce systematic setup errors in head and neck patients.^(^ ^25^ ^–^ ^26^ ^)^ In margin recipes, systematic errors are the dominant component of margin calculation. Thus, reduction of systematic error through image guidance also contributes to smaller margins.

In IMRT treatments, it is the sharp dose gradients present throughout the dose distribution that offer the potential therapeutic gains. These clinical benefits can only be accomplished if the ‘idealized’ IMRT dose distributions can be delivered as planned. In this study, dose deviations were investigated for two types of IMRT dose distributions: FO and DMPO. While in plan review, FO plans appear to be superior to DMPO plans in target coverage and conformity, FO treatments suffered larger dose deviations for targets when setup errors were accounted for. The greater sensitivity of FO dose distributions to setup uncertainties is due to the higher dose gradients (FO ${\text{CI}}_{95}$/${\text{CI}}_{100}=1.26$ vs. DMPO ${\text{CI}}_{95}/{\text{CI}}_{100}=1.35$). Required margins for FO plans are predicted to be 0.8 mm and 0.6 mm larger than the DMPO for CTV1 and CTV2, respectively. Consequently, the tight gradients which make a complex IMRT treatment plan so desirable also make them more vulnerable to setup errors. Therefore, planners should be cautious of pushing optimization algorithms too hard, as more complex dose distributions in planning does not necessarily result in better treatments.

With respect to evaluation parameters, both DVH dose metrics and conformity indices were derived for use in plan comparison only, without knowledge of the actual delivered cumulative dose. In analyzing the effects of setup errors using these planning indices, limitations were observed, specifically for the conformity indices CI and CN. When evaluating margins for radiotherapy, the target volume of interest is not consistent for the treatment (CTV) and the plan (PTV). For the conformity index parameter, the differences in volume between these alone results in index values for treatments much greater than those observed for plans. Even with very large patient misalignments, CI treatment values are still greater than 1, erroneously indicating that the target is still adequately covered. For the CN parameter, this volume difference results in poor healthy tissue conformity index and resulting CN values, of which conclusions cannot be drawn. Thus, for cumulative dose analysis, DVH dose metrics may be useful; however, CI and CN indices should not be used to prevent incorrect conclusions.

On the issue of dose evaluation methods, average fractional dose differed significantly from the cumulative dose due to setup errors. This is an important finding because of the growing interest in adaptive radiotherapy,^(^ ^27^ ^–^ ^28^ ^)^ where the average fractional dose, which is easier to calculate, is often used to estimate the actual dose received by the patient. The main reason for the differences is due to the fact that the random setup error is larger than the systematic. This means, for example, that not the same target region gets underdosed at every fraction. While the CUMU analysis method can account for this, the AVG method compounds each fractional underdosage as if they occur in the same location or systematically. Therefore, for fractionated radiotherapy (≥30 fractions), use of the average fractional dose does not represent the true cumulative dose.

A limitation of this work is that only rigid setup errors were investigated in depth. Therefore, the reduced margins recommended from this analysis are those necessary to account for rigid setup errors alone. Additional margins must be included to account for non‐rigid setup errors and anatomic changes such as tumor shrinkage. These are topics which have already been reported separately^(^ ^29^ ^–^ ^30^ ^)^ or are of interest for future study. Furthermore, the rigid patient setup errors measured in this study reflect use of an offline correction protocol which significantly reduces patient systematic errors. Thus, our results should be taken with caution as margin reduction will be different for clinics that employ different image guidance procedures or immobilization devices.

## V. CONCLUSIONS

In this study, we evaluated the dosimetric effects of measured patient setup errors on SIB HN‐IMRT treatments and found that conventional margin recipes significantly overestimate the margins needed for planning. With proposed 5 mm margins, the dose coverage delivered to each CTV exceeded that achieved in planning, and critical structure doses were only minimally impacted when measured setup errors were applied. For patients treated in our clinic under offline correction protocol, the planning margins can be safely reduced to ~1.9 mm for the primary CTV, and to ~1.5 mm for the elective CTV to account for rigid setup uncertainties. The more complex dose distributions with steeper gradients also are more sensitive to the setup errors. This study revealed the limitations in the use of conformation number and conformity index in evaluating treatment doses; however, normalized DVH dose metrics can still be used practically. Significant differences in the dose delivered to patients are observed between average fractional and cumulative dose computation methods. For fractionated radiotherapy, the average fractional dose does not represent the true cumulative dose and should only be used judiciously for treatment evaluation.
